# DDX3 is critical for female fertility via translational control in oogenesis

**DOI:** 10.1038/s41420-024-02242-6

**Published:** 2024-11-17

**Authors:** Shang-Yu Tsai, Chih-Hung Lin, Yu-Ting Jiang, Guo-Jen Huang, Haiwei Pi, Hsin-Yuan Hung, Woan-Yuh Tarn, Ming-Chih Lai

**Affiliations:** 1 Department of Biomedical Sciences, Chang Gung University, Taoyuan, Taiwan; 2 Graduate Institute of Biomedical Sciences, Chang Gung University, Taoyuan, Taiwan; 3 Master and PhD Program of Biotechnology Industry, Chang Gung University, Taoyuan, Taiwan; 4 Department of Colorectal Surgery, New Taipei Municipal Tucheng Hospital, New Taipei, Taiwan; 5 Institute of Biomedical Sciences, Academia Sinica, Taipei, Taiwan

**Keywords:** Mechanisms of disease, Infertility

## Abstract

DEAD-box RNA helicase 3 (DDX3) and its homologs play a vital role in translation initiation by unwinding secondary structures of selected mRNAs. The human *DDX3* gene is located on the sex chromosomes, so there are *DDX3X* and *DDX3Y*. DDX3X is ubiquitously expressed in almost all tissues and critical for embryonic development, whereas DDX3Y is only expressed in the testis and essential for male fertility. *Drosophila belle* (*bel*) is the single ortholog of DDX3, and mutations in *bel* cause male and female infertility. Using *Drosophila bel* mutants and *Ddx3x* conditional knockout (cKO) mice, we confirmed the pivotal role of DDX3 in female fertility and ovarian development. *Drosophila bel* mutants exhibited female infertility and immature egg chambers. Consistently, oocyte-specific *Ddx3x* knockout in mice resulted in female infertility and impaired oogenesis. We further found that immature egg chambers in *Drosophila bel* mutants and impaired follicular development in oocyte-specific *Ddx3x* cKO mice were caused by excessive apoptosis. We also identified a set of DDX3 target genes involved in oocyte meiosis and maturation and demonstrated that DDX3 is involved in their translation in human cells. Our results suggest that DDX3 is critical for female fertility via translational control in oogenesis.

## Introduction

The human DEAD-box protein DDX3 plays a crucial role in cellular RNA metabolism. DDX3 and its homologs have been implicated in multiple cellular processes, including translation initiation [1–6]. The yeast DDX3 homolog, Ded1, is required for translation of bulk mRNAs [1]. However, human DDX3 may affect the translation of only a subset of mRNAs. We previously reported that DDX3 is required for the translation of selected mRNAs that contain a long or structured 5′ UTR in human cells [3, 6]. Consistently, depletion of DDX3 in HCT116 cells represses translation of mRNAs with complex 5′ UTRs, such as those with high GC content or containing the cytosine-enriched regulator of translation (CERT) motif [5]. Given that the helicase activity of DDX3 is required for its function in translation [3], DDX3 may facilitate ribosome scanning by resolving secondary structures in the 5′ UTR of selected mRNAs.

DDX3 is a multifunctional protein implicated in a variety of biological processes, including cell cycle control [6–8], tumorigenesis and cancer progression [9–12], cell apoptosis [13–15], viral replication [16–18], innate immunity [19–21], and neurodevelopment [22–24]. The human *DDX3* genes are located on both the X and Y chromosomes, so there are *DDX3X* and *DDX3Y*. Although DDX3X and DDX3Y share ~92% of the protein sequence identity and can functionally complement each other in translation [25], they show distinct expression patterns and biological functions [26]. DDX3X is ubiquitously expressed in almost all tissues and critical for embryonic development [7, 27], whereas DDX3Y is only expressed in the testis, especially in spermatogonia and early spermatocytes, and essential for male fertility [28]. The most common male infertility cases are caused by deletions in the Y chromosome’s azoospermia factor (AZF) regions [29]. Deletion analysis of the Y chromosome revealed three common deletions: *AZFa*, *AZFb*, and *AZFc* [30]. *AZFa* deletion causes the most severe azoospermia phenotype, a complete absence of germ cells in the seminiferous tubules of the testis, and Sertoli cell-only (SCO) syndrome in humans [31]. The human *AZFa* region contains three genes: *USP9Y*, *DDX3Y*, and *UTY* [32]. *Uty* knockout in mice did not affect fertility [33]. Loss of the human *USP9Y* gene did not cause male infertility [34], suggesting that *USP9Y* is not the causative gene. It has been reported that *DDX3Y* has a higher mutation rate in SCO syndrome than the other two genes in the *AZFa* region [35]. Overexpression of DDX3Y in *AZFa*-deleted iPSCs restored germ cell formation [36]. Therefore, *DDX3Y* is regarded as the major gene in *AZFa* deletion-induced spermatogenic failure [35].

Mouse *Ddx3x* mRNA is predominantly expressed in the ovary and embryo [37], implying that Ddx3x may play a role in the ovary and embryo. Conventional knockout of *Ddx3x* in mice causes early embryonic lethality [27]. Mouse Ddx3x protein is expressed in germinal vesicle (GV) oocytes, and its expression is remarkably increased in metaphase II oocytes [7]. This finding suggests a role for mouse Ddx3x in oocyte development. *Drosophila belle* (*bel*), the single ortholog of human *DDX3*, is required for larval growth and can functionally substitute for the yeast *Ded1* in vivo [38], suggesting the functional conservation of DDX3 homologs. It has been reported that *Drosophila bel* mutants are male- and female-sterile [38]. Ectopic expression of *bel* ATPase mutants results in decreases in female fertility and ovary mass [39]. A recent study showed that DDX3 is required for ovarian development and oocyte maturation in *Locusta migratoria* [40]. These results reveal an evolutionarily conserved role of DDX3 in the development of germ cells. However, the molecular mechanisms of DDX3 in gametogenesis (spermatogenesis and oogenesis) remain largely unclear.

Here, we used *Drosophila bel* mutants and *Ddx3x* conditional knockout (cKO) mouse models to confirm the importance of DDX3 in female fertility and ovarian development. We previously identified many target mRNAs whose translation is regulated by DDX3 in HeLa cells [6]. Pathway enrichment analysis showed that ten candidate DDX3 target genes are involved in oocyte meiosis, oocyte maturation, and the gonadotropin-releasing hormone (GnRH) signaling pathway. Therefore, we assume that DDX3 is critical for female fertility via translational control in oogenesis.

## Results

### *Drosophila bel* mutants are female-sterile

**Fig. 1:**
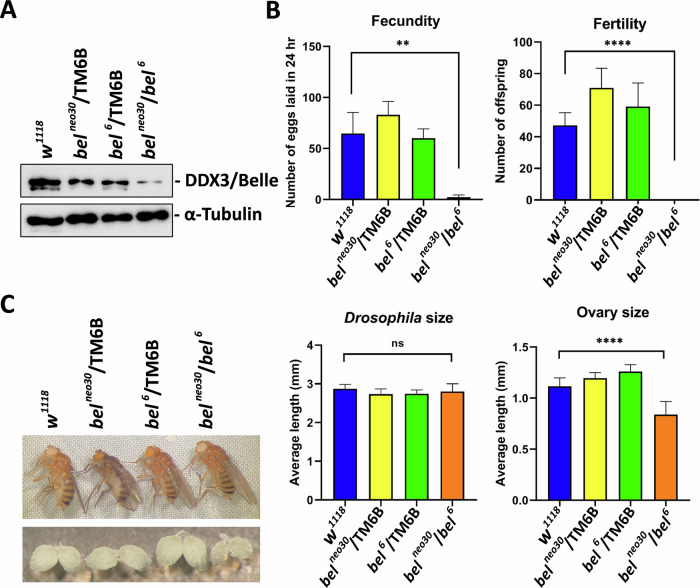
Mutations in *Drosophila bel* cause female infertility and ovarian agenesis. **A** Adult females were lysed in 1× RIPA Lysis Buffer and subjected to western blot analysis using antibodies against DDX3 and α-tubulin proteins. Detection of α-tubulin served as a loading control. **B** Three virgin females were mated with two *w*^*1118*^ males for 5 days. Fecundity was assessed by the number of eggs laid within 24 h. Fertility was evaluated by the number of offspring after 19 days of ovulation. The strain *w*^*1118*^ served as a control. Data are shown as mean and standard deviation (*n* = 10/group). **C** Adult females were fed diets containing yeast for 3 days and then dissected to collect their ovaries. Body length and ovary size were measured and recorded under a dissecting microscope. Data are shown as mean and standard deviation (*n* = 10/group). Statistical significance was determined using the Student’s *t*-test (ns not significant; **p < 0.01; ****p < 0.0001).

*Drosophila bel* is the single ortholog of human *DDX3*. It has been reported that *Drosophila bel* mutations cause male and female infertility [38, 39]. We first need to verify the experimental results. The *bel*^*neo30*^ is a hypomorphic mutation with a P-element insertion at the first intron of the *bel* gene. The *bel*^6^ is a lethal mutation with a point mutation that causes a premature stop codon. To verify whether *Drosophila bel* is required for female fertility, the *bel*^*neo30*^*/TM6B* heterozygotes and the *bel*^*6*^*/TM6B* heterozygotes were crossed to produce the trans-heterozygous allelic combination *bel*^*neo30*^*/bel*^*6*^. Western blot analysis showed that the expression of bel/DDX3 protein was markedly decreased in *Drosophila bel*^*neo30*^*/bel*^*6*^ female mutants (Fig. 1A). Compared to the *w*^*1118*^ strain and heterozygous mutations (*bel*^*neo30*^*/TM6B* and *bel*^*6*^*/TM6B*), *Drosophila bel*^*neo30*^*/bel*^*6*^ females laid a lot fewer eggs, and did not produce any viable adults (Fig. 1B). The results indicate that *Drosophila bel*^*neo30*^*/bel*^*6*^ mutants are female-sterile.

### Ovarian development is impaired in *Drosophila bel* mutants

In *Drosophila*, body size is one of the most determining traits in sexual selection [41]. The size of the *Drosophila* body was measured and recorded under a dissecting microscope. There was no significant change in the mean length of the *Drosophila* body in *bel*^*neo30*^*/bel*^*6*^ female mutants compared to the *w*^*1118*^ strain or single-allele mutations (*bel*^*neo30*^*/TM6B* and *bel*^*6*^*/TM6B*) (Fig. 1C). To understand why *Drosophila bel*^*neo30*^*/bel*^*6*^ mutants are female-sterile, we dissected adult females to observe their ovaries. An adult female has two ovaries. Notably, we observed that the size and volume of the ovaries were much smaller in *bel*^*neo30*^*/bel*^*6*^ female mutants compared to the *w*^*1118*^ strain or single-allele mutations (Fig. 1C). The results suggest that *Drosophila* bel/DDX3 plays a critical role in ovarian development.

### Apoptosis results in immature egg chambers in *Drosophila bel* mutants

**Fig. 2:**
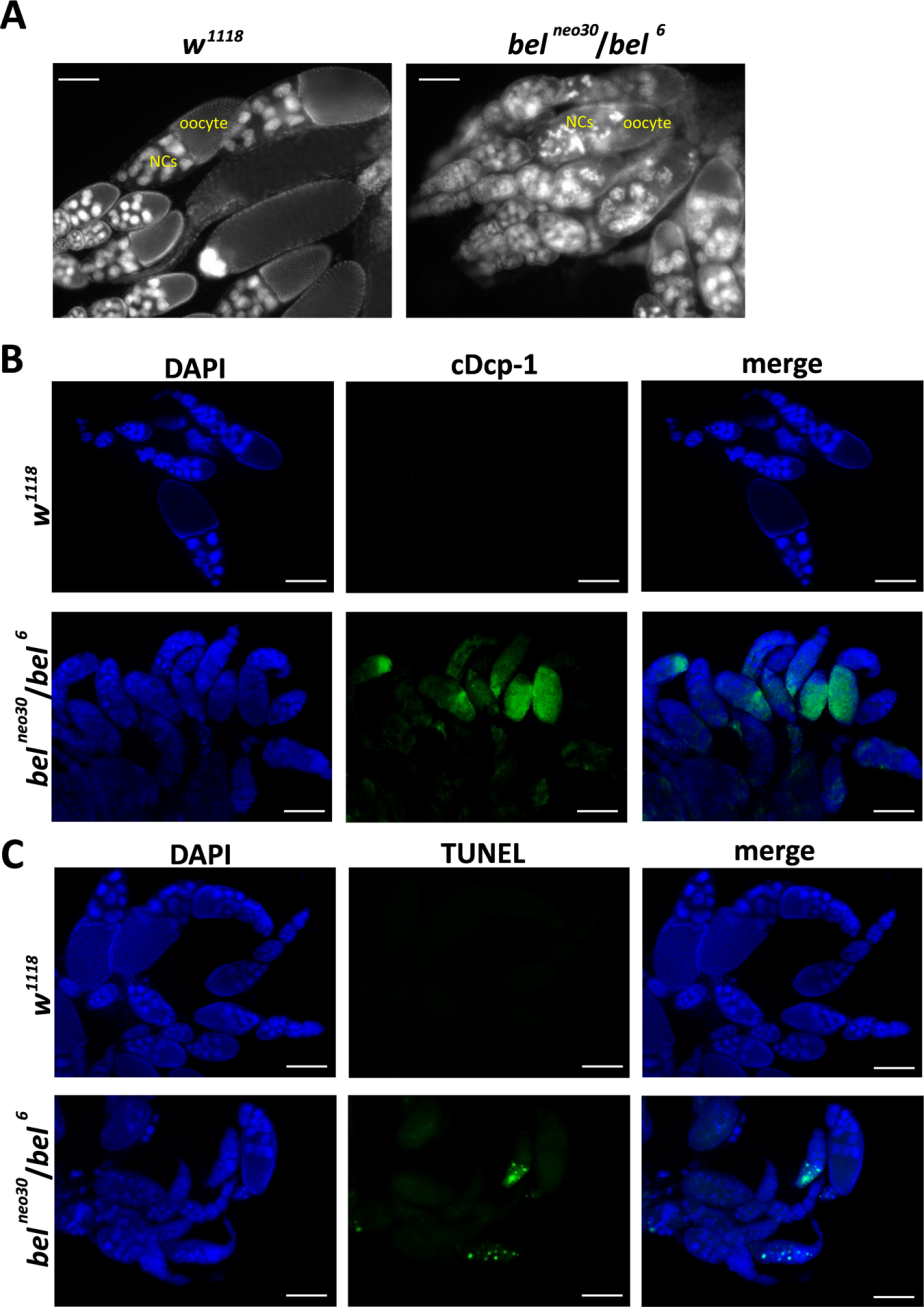
Immature egg chambers and apoptosis in *Drosophila bel*^*neo30*^*/bel*^*6*^ mutants. Adult females (*w*^*1118*^ and *bel*^*neo30*^*/bel*^*6*^) were fed diets containing yeast for 3 days and then dissected to collect their ovaries. Ovaries were fixed with 4% formaldehyde and washed with 1× PBS containing 0.3% Triton X-100. **A** DAPI staining shows the presence of nuclei in *Drosophila* ovaries. NCs: nurse cells. **B** Ovaries were permeabilized with 1% Triton X-100. Immunofluorescence staining was performed using antibodies against cleaved Dcp-1 (cDcp-1), commonly used as a marker of apoptosis in *Drosophila*. DAPI staining shows the presence of nuclei in ovaries. **C** Ovaries were permeabilized with 1% Triton X-100. TUNEL assay was performed to detect apoptotic DNA fragmentation in *Drosophila* ovaries. DAPI staining shows the presence of nuclei in ovaries. Scale bars, 100 μm.

To explore why *Drosophila bel*^*neo30*^*/bel*^*6*^ mutants lose female fertility, dissected ovaries were stained with DAPI and observed using a fluorescence microscope. We did not find mature oocytes in *bel*^*neo30*^*/bel*^*6*^ (Fig. 2A). The disruption of nuclei in nurse cells (NCs) of egg chambers was noticeable (Fig. 2A). We therefore speculate that apoptosis of egg chambers may occur in the development of the *bel*^*neo30*^*/bel*^*6*^ ovaries, thus resulting in the arrest of oocyte maturation. To test this hypothesis, we performed immunofluorescence (IF) and terminal deoxynucleotidyl transferase-mediated dUTP nick-end labeling (TUNEL) staining to detect apoptosis in *Drosophila* ovaries. Cleaved Dcp-1(cDcp-1) in *Drosophila*, a homolog of mammalian caspase 3, is a specific marker of apoptosis [42]. Apoptotic cells can be labeled with cDcp-1 and TUNEL in *Drosophila* tissues [43]. The results of IF and TUNEL staining showed that massive apoptosis of egg chambers occurs in the *bel*^*neo30*^*/bel*^*6*^ ovaries compared to the *w*^*1118*^ ovaries (Fig. 2B, C). Therefore, mutations in *Drosophila bel* resulted in apoptosis of egg chambers.

### Generation of oocyte-specific *Ddx3x* conditional knockout (cKO) mice

**Fig. 3:**
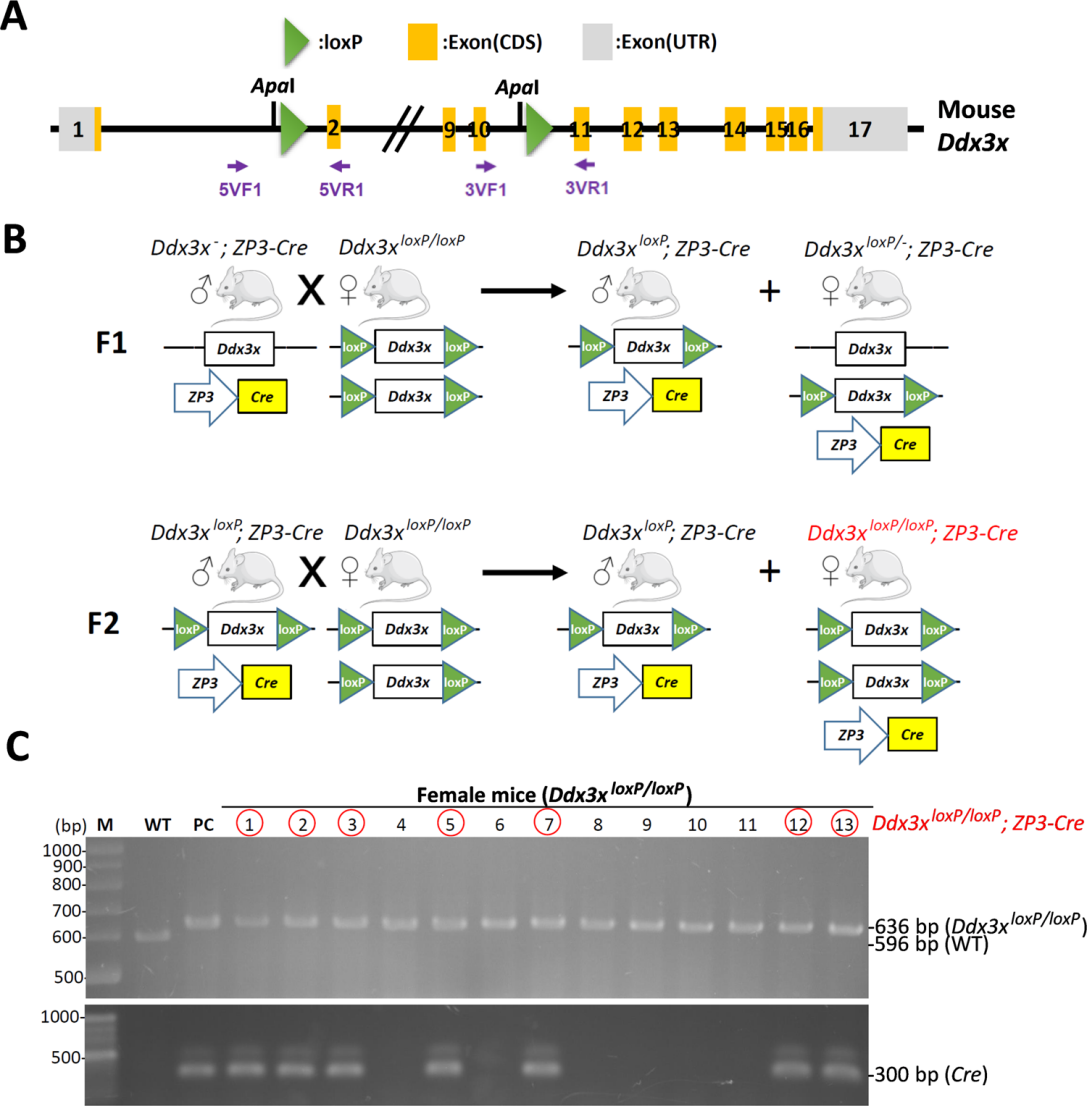
Oocyte-specific *Ddx3x* cKO mice are generated and genotyped by PCR analysis. **A** The loxP site (ATAACTTCGTATAATGTATGCTATACGAAGTTAT), followed by an *Apa*I restriction site (GGGCCC), was inserted into intron 1 and intron 10 of the *Ddx3x* gene using a CRISPR/Cas9-based gene editing strategy. The length of a PCR product with a loxP site will increase by 40 bp, and it can be cut into two fragments by the *Apa*I restriction enzyme. **B** Diagram of breeding strategy for generation of Cre-loxP-mediated oocyte-specific *Ddx3x* cKO mice. **C** Homozygous *Ddx3x*^*loxP/loxP*^ female mice were detected by PCR product (636 bp) compared to wild-type mice (596 bp). *Zp3-Cre* mice were detected by PCR product (~300 bp). Among the 13 female mice, 7 were oocyte-specific *Ddx3x* cKO mice (*Ddx3x*^*loxP/loxP*^*; Zp3-Cre*) (red circle).

As mentioned above, conventional knockout of *Ddx3x* in mice results in early embryonic lethality [27]. We therefore used oocyte-specific *Ddx3x* cKO mice to study the importance of Ddx3x in oogenesis. A *Ddx3x* cKO mouse model, in which the *Ddx3x* gene is flanked with loxP sites using a CRISPR/Cas9-based gene editing, has been established (Fig. 3A). According to the Cre-loxP system [44], gene deletion can be precisely controlled by spatiotemporally defined Cre recombinase expression [45]. A transgenic mouse line *Zp3-Cre* in which Cre recombinase expression is controlled by the promoter of the zona pellucida 3 (*Zp3*) gene, which is exclusively expressed in the growing oocytes [46]. The *Zp3-Cre* line is used to delete the specific gene in oocytes [47]. To generate oocyte-specific *Ddx3x* cKO female mice (*Ddx3x*^*loxP/loxP*^*; Zp3-Cre*), heterozygous *Ddx3x*^*loxP*^ mice were inbred to produce homozygous *Ddx3x*^*loxP/loxP*^ female mice and then crossed with *Zp3-Cre* male mice (Fig. 3B). Oocyte-specific *Ddx3x* cKO female mice were confirmed by PCR-based genotyping (Fig. 3C).

### Female infertility in oocyte-specific *Ddx3x* cKO mice

**Fig. 4:**
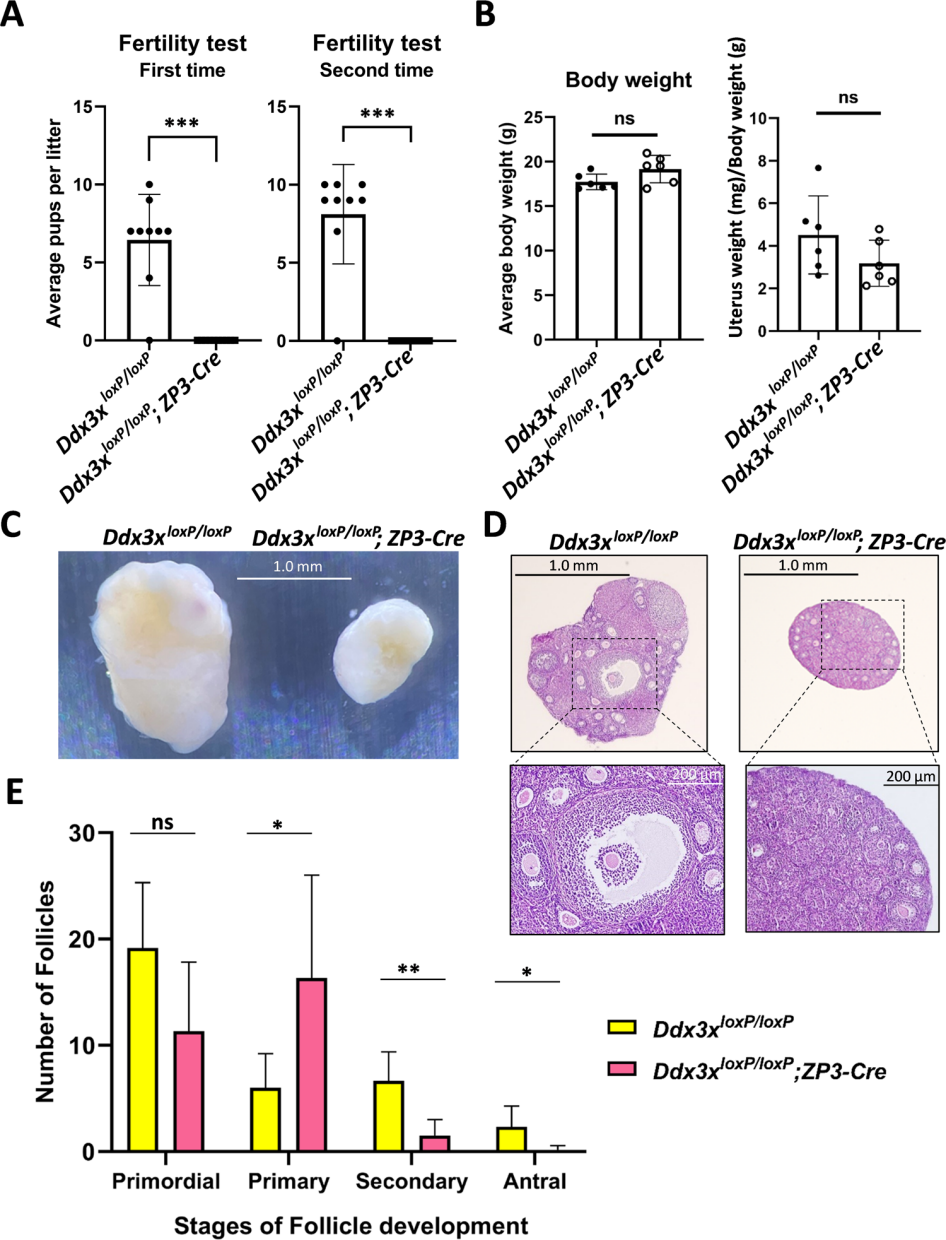
Female infertility and impaired ovarian development in oocyte-specific *Ddx3x* cKO mice. **A** Ten-week-old *Ddx3x*^*loxP/loxP*^ female mice (*n* = 9) and *Ddx3x*^*loxP/loxP*^*; Zp3-Cre* female mice (*n* = 10) were mated with wild-type C57BL/6 male mice for 10 days (1 female:1 male). Females were then separated from the males and allowed to rest for 21 days, the average gestation period in mice. The number of pups was counted after birth. Fertility tests were carried out twice. Data are shown as mean ± SD. **B** Ten-week-old *Ddx3x*^*loxP/loxP*^ female mice (*n* = 6) and *Ddx3x*^*loxP/loxP*^*; Zp3-Cre* female mice (*n* = 6) were dissected to collect their uteruses (with ovaries). The weight of the body and uterus (with ovaries) was measured and recorded. The ratio of uterus weight to body weight of mice was calculated. Data are shown as mean ± SD. **C** Experimental mice are as described in (**B**). Mouse ovaries were observed under a dissecting microscope. Representative images of mouse ovaries were shown. **D** Experimental mice are as described in (B). Mouse ovaries were fixed in formalin, embedded in paraffin, and then sectioned at a thickness of 5 μm. Ovarian sections were stained with hematoxylin and eosin (H&E). Representative images of H&E-stained ovarian sections were shown. **E** Experimental mice were prepared and treated as described in (**D**). The number of follicles in the ovarian sections at different stages (primordial, primary, secondary, and antral) was counted. The bar graph shows the number of follicles at different stages in the ovaries of oocyte-specific *Ddx3x* cKO mice (*Ddx3x*^*loxP/loxP*^*; Zp3-Cre*) compared to control mice (*Ddx3x*^*loxP/loxP*^). Data are shown as mean and standard deviation (*n* = 6/group). Statistical significance was determined using the Student’s *t*-test (ns not significant; *p < 0.05; **p < 0.01; ***p < 0.001).

To examine whether oocyte-specific deletion of *Ddx3x* affects female fertility, the fertility test was carried out twice to reduce experimental errors. The number of offspring produced by mating 10-week-old *Ddx3x*^*loxP/loxP*^ or *Ddx3x*^*loxP/loxP*^*; Zp3-Cre* female mice with normal C57BL/6 male mice were counted. Male mice were randomly assigned to female mice. A female mouse is considered to be fertile if she gives birth to pups. The number of offspring from each pregnancy was recorded after birth. The average litter size was evaluated in *Ddx3x*^*loxP/loxP*^; *Zp3-Cre* female mice compared to *Ddx3x*^*loxP/loxP*^ female mice. As expected, *Ddx3x*^*loxP/loxP*^*; Zp3-Cre* female mice did not give birth to any pups, whereas *Ddx3x*^*loxP/loxP*^ female mice had an average of 7–8 pups per litter (Fig. 4A). The results indicated that oocyte-specific *Ddx3x* cKO mice are female-sterile.

### Follicular development is impaired in oocyte-specific *Ddx3x* cKO mice

There was no significant difference in appearance and body weight between *Ddx3x*^*loxP/loxP*^ and *Ddx3x*^*loxP/loxP*^*; Zp3-Cre* female mice (Fig. 4B). To understand why oocyte-specific *Ddx3x* cKO mice lose female fertility, we dissected *Ddx3x*^*loxP/loxP*^ and *Ddx3x*^*loxP/loxP*^*; Zp3-Cre* female mice to observe their ovaries. The weight of the uterus (with ovaries) was measured and recorded. The uterus index, defined as the ratio of uterus weight to body weight of mice, was calculated and compared to the control group. There was only a slight decrease but no significant difference in the uterus index of *Ddx3x*^*loxP/loxP*^*; Zp3-Cre* female mice compared to *Ddx3x*^*loxP/loxP*^ female mice (Fig. 4B). In contrast, the size of the ovaries of *Ddx3x*^*loxP/loxP*^*; Zp3-Cre* female mice were much smaller than that of *Ddx3x*^*loxP/loxP*^ female mice (Fig. 4C). The ovaries of *Ddx3x*^*loxP/loxP*^ and *Ddx3x*^*loxP/loxP*^*; Zp3-Cre* female mice were collected, fixed in formalin, and embedded in paraffin for long-term storage. Follicular development was observed in hematoxylin and eosin (H&E)-stained ovarian sections. Notably, it is difficult to find secondary and antral follicles in the ovaries of *Ddx3x*^*loxP/loxP*^*; Zp3-Cre* female mice (Fig. 4D). The ovarian tissue of *Ddx3x*^*loxP/loxP*^*; Zp3-Cre* female mice showed the absence of corpus luteum (Fig. 4D). The accumulation of primary follicles in oocyte-specific *Ddx3x* cKO female mice suggested that deletion of *Ddx3x* inhibits the primary to secondary follicle transition (Fig. 4E). The results supported that Ddx3x is required for the development of ovaries and mature follicles in female mice. However, the molecular mechanisms of DDX3 in ovarian development and oogenesis remain to be further studied.

### Extensive apoptosis occurs in the ovarian follicles of **o**ocyte-specific *Ddx3x* cKO female mice

**Fig. 5:**
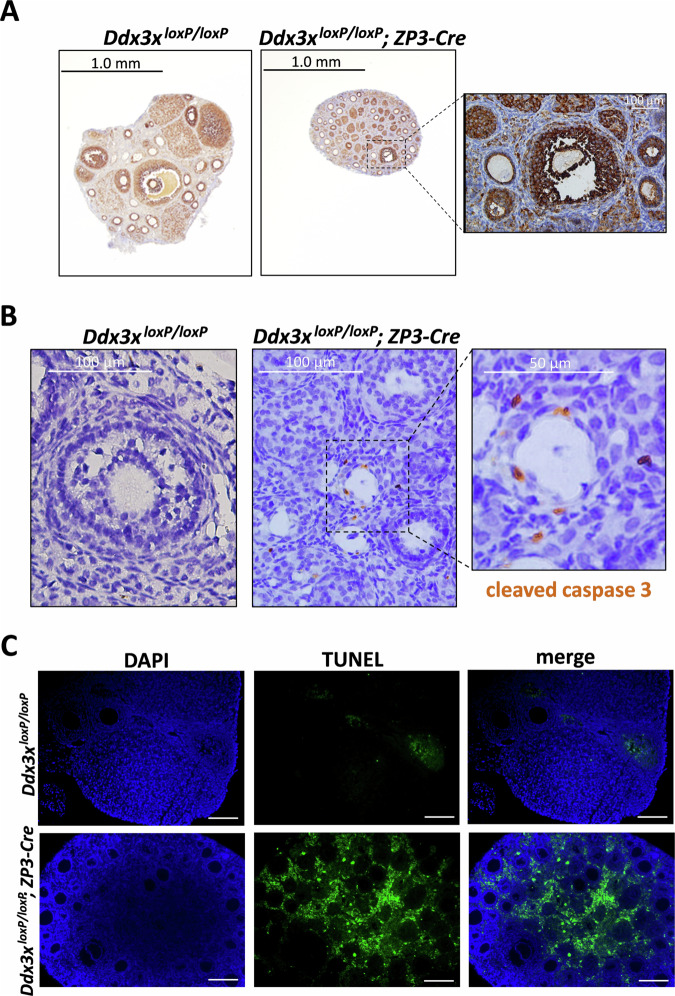
Impaired oogenesis and extensive apoptosis occur in the ovaries of oocyte-specific *Ddx3x* cKO mice. 10-week-old *Ddx3x*^*loxP/loxP*^ female mice (*n* = 6) and *Ddx3x*^*loxP/loxP*^*; Zp3-Cre* female mice (*n* = 6) were dissected to collect their ovaries. Mouse ovaries were fixed in formalin, embedded in paraffin, and then sectioned at a thickness of 5 μm. **A** Ovarian sections were processed for immunohistochemistry (IHC) to stain endogenous mouse Ddx3x. Representative images of IHC-stained ovarian sections were shown. An antral follicle and nearby degenerated oocytes were enlarged in *Ddx3x*^*loxP/loxP*^*; Zp3-Cre* ovarian sections. **B** Ovarian sections were processed for immunohistochemistry (IHC) to stain cleaved caspase 3. Representative images of IHC-stained ovarian sections were shown. Brown signals were frequently observed in *Ddx3x*^*loxP/loxP*^*; Zp3-Cre* ovarian sections. **C** TUNEL assay was performed to detect apoptotic DNA fragmentation in mouse ovarian sections. DAPI staining shows the presence of nuclei in mouse ovaries. Scale bars, 100 μm.

We also performed immunohistochemistry (IHC) staining with DDX3 antibody to detect oocyte-specific knockout of *Ddx3x* in the ovaries of *Ddx3x*^*loxP/loxP*^*; Zp3-Cre* female mice. However, the IHC staining of Ddx3x in ovarian oocytes is too light to distinguish between *Ddx3x*^*loxP/loxP*^ and *Ddx3x*^*loxP/loxP*^*; Zp3-Cre* female mice (Fig. 5A). Among the observed ovarian sections of oocyte-specific *Ddx3x* cKO female mice, only one antral follicle was seen (Fig. 5A). Notably, oocyte collapse/degeneration was observed in the ovaries of oocyte-specific *Ddx3x* cKO female mice (Fig. 5A). The phenotype of oocyte collapse/degeneration suggested that cells may undergo apoptosis. To examine whether oocyte-specific deletion of *Ddx3x* results in apoptosis in the ovarian follicles, we performed IHC and TUNEL staining to detect apoptosis in mouse ovarian sections. Cleaved caspase 3 is a specific marker of apoptosis in mouse oocytes [48]. IHC staining with cleaved caspase 3 antibody and TUNEL assay were performed to detect apoptotic cells in mouse ovarian sections. The results of cleaved caspase 3 and TUNEL staining showed that extensive apoptosis occurs in ovarian sections of *Ddx3x*^*loxP/loxP*^*; Zp3-Cre* female mice compared to *Ddx3x*^*loxP/loxP*^ female mice (Fig. 5B, C). Therefore, loss of *Ddx3x* in mouse oocytes results in a female infertility phenotype due to apoptosis of ovarian follicles.

### DDX3-regulated candidate genes are involved in oogenesis

Because it is difficult to collect mouse oocytes from oocyte-specific *Ddx3x* cKO female mice, we re-analyzed a set of translational targets of DDX3 that were previously identified in HeLa cells [6]. To gain insight into the biological functions of DDX3, pathway enrichment analysis was performed using the DAVID Bioinformatics Resources software (https://david.ncifcrf.gov/) to map genes to biological pathways defined by the Kyoto Encyclopedia of Genes and Genomes (KEGG) database. The result showed that a set of candidate genes are involved in oocyte meiosis, progesterone-mediated oocyte maturation, and the GnRH signaling pathway (Table 1). These candidate DDX3 target genes include *APC1, CCNE1, P38γ, B56β, PP2Aβ, PKACα, FBXW11, RAC1, P38β*, and *GNAS* (Table 1). Since these candidate genes have been implicated in oocyte meiosis and maturation (Table 2), we speculate that DDX3 has a role in oogenesis.

**Table 1:** Pathway enrichment analysis of candidate DDX3 target genes involved in oogenesis.

KEGG pathway	*p* Value	Genes
Oocyte meiosis	0.0045	*APC1*, *CCNE1*, *P38γ*, *B56β*, *PP2Aβ, PKACα*, *FBXW11*
Progesterone-mediated oocyte maturation	0.0357	*APC1*, *RAC1, P38γ*, *PKACα*, *P38β*
GnRH signaling pathway	0.0411	*P38γ*, *PKACα*, *P38β, GNAS*

**Table 2:** DDX3-regulated candidate genes involved in meiosis and oogenesis.

Genes	Effects on oogenesis	References
*APC1*	Triggers the metaphase to anaphase transition in mitosis and meiosis	[59]
*CCNE1*	Acts as an important regulator of mitosis and meiosis	[60]
*P38γ*	Promotes meiotic G2/M transition in *Xenopus* oocytes	[61]
*B56β*	Regulates chromosome congression during meiosis I	[62]
*PP2Aβ*	Is critical for regulating oocyte meiosis	[63]
*PKACα*	Regulates the resumption of meiosis in mammalian oocytes	[64]
*FBXW11*	Plays a vital role in gametogenesis and embryonic development	[65]
*RAC1*	Regulates meiotic spindle stability and anchoring to the cortex	[66]
*P38β*	Is required for egg asymmetric development in *Drosophila* oogenesis	[67]
*GNAS*	Maintains meiotic arrest in the mouse follicle	[68]

### DDX3 regulates the protein expression of oogenesis-associated genes in HeLa cells

**Fig. 6:**
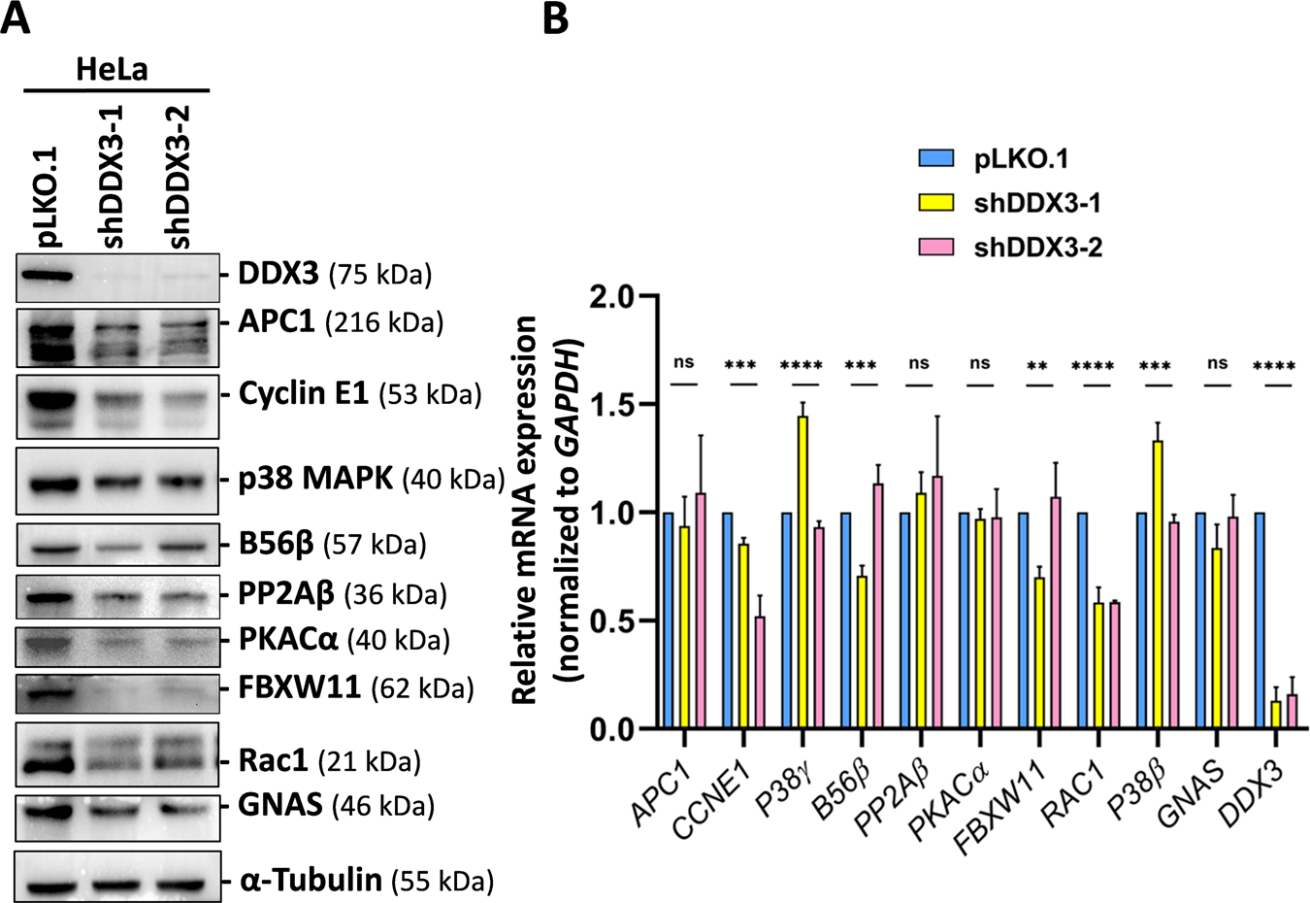
DDX3 regulates the protein expression of candidate target genes involved in oogenesis in HeLa cells. HeLa cells were transduced with the empty lentiviral vector (pLKO.1) or the pLKO.1 vector expressing DDX3 shRNAs (shDDX3-1 and shDDX3-2). Cells were harvested for analysis at day 3 post-transduction. **A** Western blot analysis was performed using antibodies against DDX3, APC1, cyclin E1, p38 MAPK, B56β, PP2Aβ, PKACα, FBXW11, Rac1, GNAS, and α-tubulin proteins. Detection of α-tubulin served as a loading control. **B** The expression of *APC1, CCNE1, P38γ, B56β, PP2Aβ, PKACα, FBXW11, RAC1, P38β, GNAS, DDX3*, and *GAPDH* mRNAs were detected by quantitative real-time RT-PCR. The bar graph shows the relative mRNA levels normalized to *GAPDH* as mean and standard deviation from three independent experiments. Statistical significance was tested by one-way ANOVA (ns not significant; **p < 0.01; ***p < 0.001; ****p < 0.0001).

We identified a set of DDX3 target genes involved in oogenesis from HeLa cells (Table 1). Next, we evaluated whether DDX3 regulates their protein expression. Using short hairpin RNAs (shDDX3-1 and shDDX3-2), we observed that the level of DDX3 protein was considerably reduced in HeLa cells (Fig. 6A). The protein levels of APC1, cyclin E1, PP2Aβ, PKACα, FBXW11, Rac1, and GNAS were prominently decreased in DDX3 knockdown HeLa cells compared to mock-treated cells (pLKO.1) (Fig. 6A). However, the level of p38 MAPK and B56β proteins were moderately decreased. The results suggest that DDX3 positively regulates the expression of APC1, cyclin E1, p38 MAPK, B56β, PP2Aβ, PKACα, FBXW11, Rac1, and GNAS proteins in HeLa cells. To examine whether the knockdown of DDX3 affects the expression of *APC1, CCNE1, P38γ, B56β, PP2Aβ, PKACα, FBXW11, RAC1, P38β*, and *GNAS* mRNAs, we used quantitative real-time RT-PCR to detect the mRNA levels. The results showed that the expression of *DDX3* mRNA was dramatically depleted by shDDX3-1 and shDDX3-2 in HeLa cells (Fig. 6B). There was no significant change in the mRNA levels of *APC1, PP2Aβ, PKACα*, and *GNAS* in DDX3 knockdown HeLa cells compared to mock-treated cells (Fig. 6B). However, *CCNE1* and *RAC1* mRNAs were significantly decreased in DDX3 knockdown HeLa cells, suggesting that DDX3 knockdown may affect the transcription or stability of *CCNE1* and *RAC1* mRNAs. Since depletion of DDX3 does not decrease the mRNA expression of most candidate DDX3 target genes, DDX3 may promote their expression at the translation level.

### Translational efficiency of *CCNE1, P38γ, B56β, PP2Aβ, PKACα, FBXW11, RAC1, P38β*, and *GNAS* mRNAs is decreased in DDX3 knockdown 293T cells

**Fig. 7:**
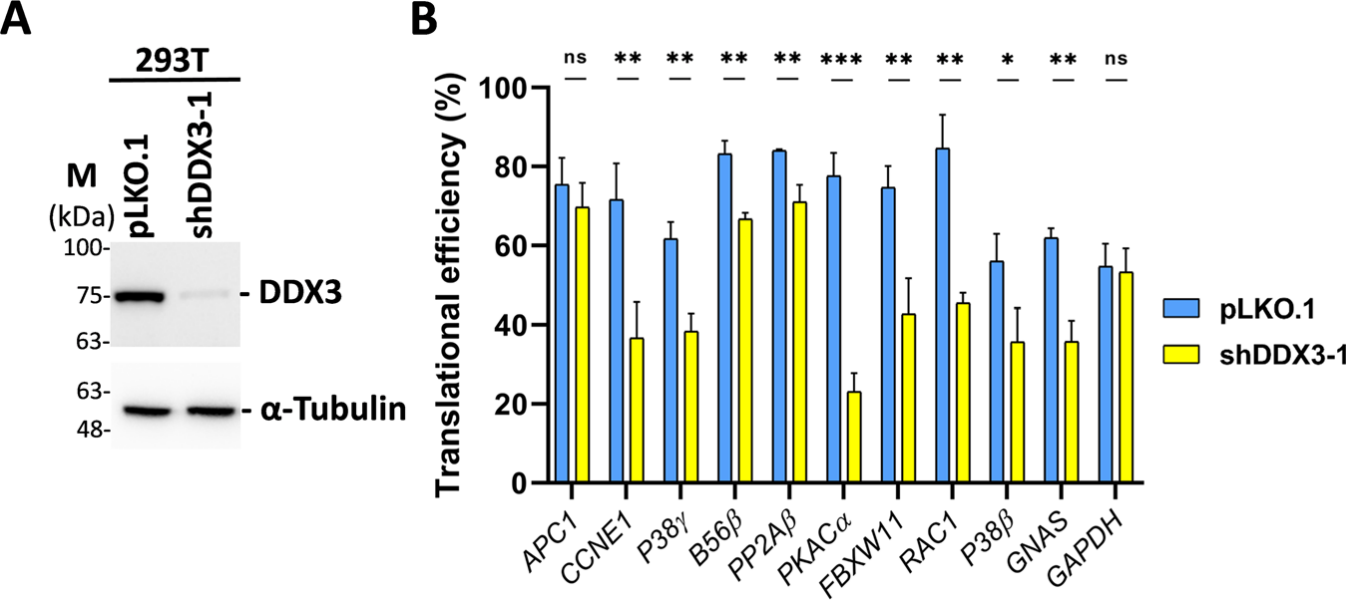
DDX3 regulates translation of candidate target genes involved in oogenesis in 293T cells. 293T cells were transduced with the empty lentiviral vector (pLKO.1) or the pLKO.1 vector expressing shDDX3-1. Cells were harvested for analysis at day 3 post-transduction. **A** Western blot analysis was performed using antibodies against DDX3 and α-tubulin proteins. Detection of α-tubulin served as a loading control. **B** Cytoplasmic extracts were loaded on a linear 15-40% sucrose gradient ultracentrifugation. Total RNA was extracted from each fraction for analysis. Polysome profile analysis and quantitative real-time RT-PCR were performed to assess translational efficiency of *APC1, CCNE1, P38γ, B56β, PP2Aβ, PKACα, FBXW11, RAC1, P38β, GNAS*, and *GAPDH* mRNAs in DDX3 knockdown 293T cells (shDDX3-1) compared to control cells (pLKO.1). Detection of *GAPDH* mRNA served as a negative control. The bar graph shows the changes in translational efficiency as mean and standard deviation from three independent experiments. Statistical significance was determined using the Student’s *t*-test (ns not significant; *p < 0.05; **p < 0.01; ***p < 0.001).

To verify whether DDX3 regulates the translation of its target mRNAs, we performed sucrose gradient sedimentation and polysome profile analysis to evaluate the translational status of DDX3-knockdown and mock-treated 293T cells. As in HeLa cells (Fig. 6), shDDX3-1 significantly downregulated the protein level of DDX3 in 293T cells (Fig. 7A). Translational efficiency of a transcript was defined as the ratio of the mRNA abundance in the polysome fractions to the mRNA abundance in the total fractions [49]. We assessed translational efficiency of *APC1, CCNE1, P38γ, B56β, PP2Aβ, PKACα, FBXW11, RAC1, P38β, GNAS*, and *GAPDH* mRNAs by polysome profiling and quantitative real-time RT-PCR. Among all transcripts examined, only *APC1* and *GAPDH* mRNAs were not significantly decreased in DDX3 knockdown 293T cells compared to mock-treated cells (Fig. 7B). Therefore, DDX3 may regulate translation of *CCNE1, P38γ, B56β, PP2Aβ, PKACα, FBXW11, RAC1, P38β*, and *GNAS* mRNAs in 293T cells. Since depletion of DDX3 does not significantly affect translational efficiency of *APC1* mRNA, the downregulation of APC1 protein in DDX3 knockdown 293T cells may result from protein destabilization. However, it remains to be studied further.

### DDX3 may facilitate the translation of oogenesis-related target mRNAs by resolving their 5′ UTRs

**Fig. 8:**
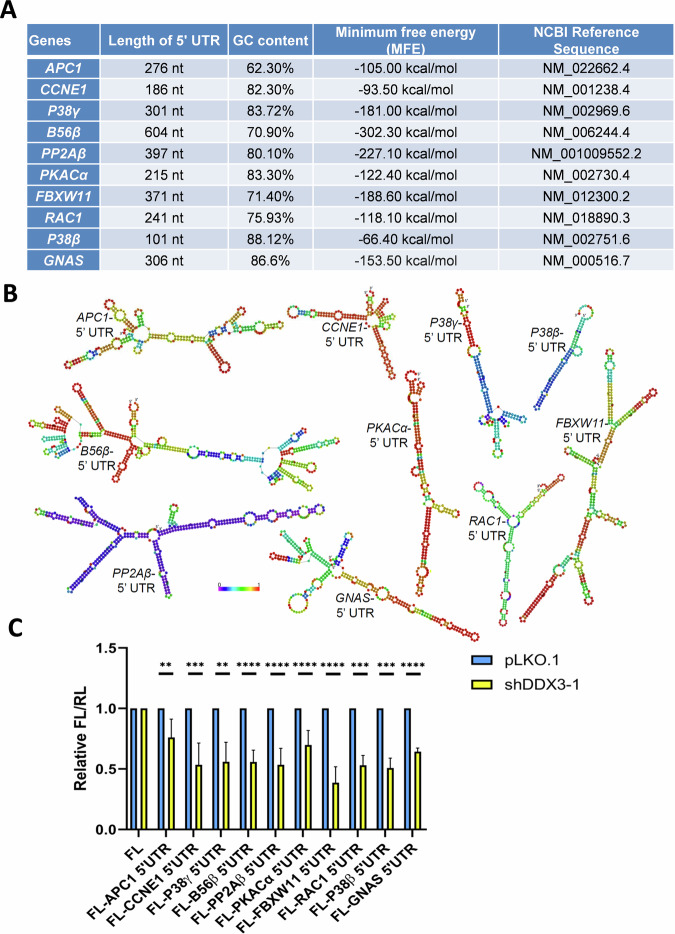
DDX3 facilitates translation of candidate target mRNAs that contain complex 5′ UTRs. **A** The 5′ UTR sequences of *APC1, CCNE1, P38γ, B56β, PP2Aβ, PKACα, FBXW11, RAC1, P38β*, and *GNAS* mRNAs obtained from the NCBI Reference Sequence (RefSeq) database were analyzed. The length, GC content, and minimum free energy (MFE) of the 5′ UTR sequences were indicated. **B** Secondary structures within the 5′ UTRs were predicted using the RNAfold web server. The nucleotides are colored according to their probabilities in the structure. **C** 293T cells were transduced with the empty lentiviral vector (pLKO.1) or the pLKO.1 vector expressing shDDX3-1. After 48 h, 293T cells were co-transfected with firefly luciferase (Fluc) reporters containing the 5′ UTRs of *APC1, CCNE1, P38γ, B56β, PP2Aβ, PKACα, FBXW11, RAC1, P38β*, or *GNAS* mRNAs in combination with the control pRL-SV40 vector encoding the *Renilla* luciferase (Rluc). 293T cells were lysed for analysis at 24 h post transfection. The Fluc activity was normalized for each transfectant to that of the Rluc control. The bar graph shows the relative Fluc/Rluc activities in DDX3 knockdown 293T cells (shDDX3-1) compared to control cells (pLKO.1). Data are shown as mean and standard deviation from three independent experiments. Statistical significance was determined using the Student’s *t*-test (**p < 0.01; ***p < 0.001; ****p < 0.0001).

We have reported that DDX3 is required for efficient translation of selected mRNAs that contain a long or structured 5′ UTR [3, 6]. Computational analysis showed that oogenesis-related DDX3 target mRNAs contain long and GC-rich 5′ UTRs that are likely to form stable secondary structures; their average length is 300 nucleotides (nt), with a GC content of 78.5% (Fig. 8A). In contrast, the average length of 5′ UTRs in randomly selected mRNAs is only about 246 nt, with a GC content of 63% [6]. Secondary structures were predicted using the RNAfold web server in ViennaRNA Web Services (http://rna.tbi.univie.ac.at/cgi-bin/ RNAWebSuite/RNAfold.cgi). The RNAfold program was used to calculate the minimum free energy (MFE) of RNA secondary structures (Fig. 8A). The secondary structures within the 5′ UTRs of candidate DDX3 target mRNAs are shown in Fig. 8B. To determine whether DDX3 participates in ribosome scanning by resolving secondary structures in the 5′ UTR of candidate target mRNAs, we performed the dual-luciferase reporter assay in 293T cells. Firefly luciferase (Fluc) reporter genes containing the 5′ UTRs of the selected candidates were constructed. A *Renilla* luciferase (Rluc) reporter with an unstructured 5′ UTR was co-transfected with the Fluc reporter as an internal control. The translation activity of the reporter mRNAs was assessed using the firefly to *Renilla* luciferase activity ratio (Fluc/Rluc). The results showed that the relative Fluc/Rluc activity of reporters was significantly decreased in DDX3 knockdown 293T cells compared to mock-treated cells (Fig. 8C). Therefore, DDX3 may facilitate translation of oogenesis-related target mRNAs by resolving secondary structures within the 5′ UTRs.

## Discussion

Although the human *DDX3Y* gene is widely transcribed in various tissues, the DDX3Y protein is only detected in male germ cells (spermatogonia) [26]. Loss of the *DDX3Y* gene is the most likely cause of the SCO syndrome, observed in *AZFa* deletion-induced spermatogenic failure [26, 35]. The SCO syndrome is one of the most common causes of male infertility, and the patients have very low or absent spermatogenesis (oligozoospermia and azoospermia). More recently, DDX3Y was identified as the critical spermatogenic factor in *AZFa* deletion-induced azoospermia [50]. Infertility patients with loss-of-function mutations in the *DDX3Y* gene presented the SCO phenotype, suggesting that DDX3Y is essential for spermatogenesis and male fertility [50]. However, the molecular mechanisms by which DDX3Y functions in spermatogenesis remain largely unclear.

Here, we identified several DDX3-regulated target genes involved in oocyte meiosis and maturation (Tables 1 and 2), suggesting a role for DDX3 in oogenesis. These DDX3-regulated target genes may act directly on oocyte meiosis or play a regulatory role during the processes of oogenesis. Meiosis occurs during the process of spermatogenesis and oogenesis. It is conceivable that loss of DDX3Y may affect sperm meiosis and maturation (spermatogenesis). This study used *Drosophila bel* mutants and oocyte-specific *Ddx3x* cKO mice to determine that DDX3X is required for female fertility and ovarian development. *Drosophila bel* is the single ortholog of human *DDX3*, and *bel* mutations cause male and female infertility [38]. Similarly, the ortholog of *DDX3* in *Locusta migratoria* is ubiquitously expressed in various tissues with a high abundance in the testis and ovary [40]. Depletion of *LmDDX3* also blocked ovarian development and oocyte maturation [40]. In mice, there are three homologs of the DDX3 subfamily: *Ddx3x*, *Ddx3y*, and *Pl10*. Mouse Ddx3x exhibits 99% similarity to human DDX3X [7]. Mouse Ddx3x is predominantly expressed in the ovary and embryo [37], suggesting that Ddx3x is required for ovarian and embryonic development [27]. Interestingly, the expression of mouse Ddx3x protein is remarkably increased in metaphase II oocytes [7]. This finding is consistent with the requirement of Ddx3x for oocyte meiosis and oogenesis.

Recently, *Ddx3y* KO male mice were generated using CRISPR/Cas9 but showed normal spermatogenesis [51]. It was proposed that mouse *Ddx3y* may be dispensable in germ cells for male fertility. Nevertheless, the mouse contains an autosomal homolog of *DDX3*, *Pl10*, which is thought to be a pseudogene in humans [52]. Mouse Pl10 is predominantly expressed in meiotic spermatocytes [37]. Pl10-deficient male mice were shown to be infertile [53], suggesting a role for Pl10 in spermatogenesis. Pl10 may replace the role of Ddx3y during spermatogenesis in mice.

Although mutations in the *bel* gene do not affect the size of the *Drosophila* body, the size and volume of the ovaries are obviously reduced in *Drosophila bel* mutants compared to the control group (Fig. 1C). The results indicate that *Drosophila* bel/DDX3 plays a critical role in ovarian development. Consistently, oocyte-specific deletion of *Ddx3x* in mice does not significantly affect the body weight and uterus index (Fig. 4B). The size of the ovaries is obviously reduced in oocyte-specific *Ddx3x* cKO mice compared to the control group (Fig. 4C). It is surprised that only deletion of *Ddx3x* from oocytes results in impaired ovarian development, indicating the importance of maintaining oocyte integrity for the development of follicles and ovaries. Oocyte-specific deletion of *Ddx3x* causes extensive apoptosis in oocytes and adjacent granulosa cells (Fig. 5). This result demonstrates that precise communication between oocytes and granulosa cells is vital for follicle and ovarian development.

Although DDX3 can bind to most mRNAs, only a subset of mRNAs is predominantly affected by DDX3 knockdown at the translation level [5, 6]. DDX3 prefers to bind to 5′ UTRs of mRNAs and 18S rRNA [54], supporting its role in ribosome scanning during translation initiation. DDX3 regulates translation of mRNAs with complex 5′ UTRs, such as high GC content [3, 5, 6], cytosine-enriched regulator of translation (CERT) motif [5], and upstream open reading frame (uORF) [55]. Although less prevalent than in the 5′ UTR, DDX3 also binds to the coding sequence (CDS) and the 3′ UTR of mRNAs [56, 57]. It has been recently reported that DDX3 target mRNAs have high GC content in the CDS [5]. This may explain why translational efficiency of *PKACα* mRNA decreased significantly in DDX3 knockdown cells (Fig. 7B). However, the Fluc reporter with PKACα 5′ UTR is less sensitive to DDX3 knockdown (Fig. 8C). There is a high GC region at the beginning of the CDS of *PKACα* mRNA, and it may also involve DDX3-mediated translational control. DDX3 was recently shown to control translation of specific mRNAs through the 3′ UTR [56, 57]. Therefore, DDX3-mediated translational control may have different mechanisms.

DDX3 was identified as a component of an anti-apoptotic protein complex associated with death receptors [58]. Loss of *Ddx3x* results in cell cycle arrest and apoptosis in mouse embryos [27], suggesting a role for DDX3 in anti-apoptosis. DDX3 protects cells from sanguinarine-induced intrinsic apoptosis in HeLa cells [14]. After sanguinarine treatment, DDX3 upregulates the expression of anti-apoptotic gene (*Bcl-xL*) and downregulates the expression of pro-apoptotic genes (*CASP3* and *BAX*) [14]. Consistent with previous studies, we also found that DDX3 interacts with USP9X and exerts anti-apoptotic effects by deubiquitinating and stabilizing MCL1 [15]. Depletion of DDX3 results in increased cell apoptosis [15], supporting a role for DDX3 in anti-apoptosis. DDX3 may activate the anti-apoptotic *Bcl-xL* gene and repress the pro-apoptotic *CASP3* and *BAX* genes through transcriptional or translational regulation. DDX3 also stabilizes anti-apoptotic MCL1 protein through USP9X-mediated MCL1 deubiquitination [15]. However, the molecular mechanism of DDX3 in anti-apoptosis remains to be further studied.

## Materials and methods

### Drosophila stocks

*Drosophila bel* mutants *bel*^*neo30*^ and *bel*^*6*^ were obtained from the Bloomington *Drosophila* Stock Center (BDSC) at Indiana University. Dr. Haiwei Pi provided the control strain *w*^*1118*^. All fly stocks were maintained and raised at 25 °C.

### Western blot analysis

Proteins were transferred onto a polyvinylidene difluoride (PVDF) transfer membrane (Millipore). Protein blots were blocked with 3% skim milk in TBST (100 mM Tris–HCl [pH 7.6], 150 mM NaCl, and 0.1% Tween-20) at room temperature for 1 h. The primary antibodies included affinity-purified rabbit anti-DDX3 (0.1 μg/ml) [3], mouse anti-α-tubulin (0.2 μg/ml; sc-32293, Santa Cruz Biotechnology), rabbit anti-APC1 (1:1000 dilution; #13329, Cell Signaling), mouse anti-cyclin E1 (0.2 μg/ml; sc-247, Santa Cruz Biotechnology), rabbit anti-p38 MAPK (1:1000 dilution; #8690, Cell Signaling), mouse anti-PP2A-B56-β (0.4 μg/ml; sc-515676, Santa Cruz Biotechnology), rabbit anti-PP2Aβ (1:1000 dilution; GTX101690, GeneTex), rabbit anti-PKACα (1:1000 dilution; GTX104934, GeneTex), rabbit anti-FBXW11 (1:1000 dilution; GTX33193, GeneTex), mouse anti-Rac1 (1:1000 dilution; 05-389, Millipore), and rabbit anti-GNAS (1:1000 dilution; GTX113200, GeneTex). Blots were incubated with primary antibodies in the blocking buffer at room temperature for 2 h, followed by horseradish peroxidase (HRP)-conjugated secondary antibodies at room temperature for 2 h. Signals were detected using Immobilon Western chemiluminescent HRP substrate (Millipore), and images from autoradiograms were captured with the ChemiDoc touch imaging system (Bio-Rad).

### ***Drosophila*** fecundity and fertility tests

Three young virgin females (0–1 day old) were mated with two *w*^*1118*^ males for five days. After mating, females were transferred to an agar plate (9-cm Petri dish) for the fecundity test. After 24 h of ovulation, the number of eggs was observed and calculated with a dissecting microscope. For the fertility test, females were transferred to a fresh culture tube after mating. Adult females were removed 24 h after spawning, and eggs were incubated for 19 days. The number of adult offspring was counted.

### DAPI and immunofluorescence staining of ***Drosophila*** ovaries

Adult females were anesthetized by CO_2_ and dissected to get their ovaries in cold 1× PBS. Ovaries were fixed with 4% formaldehyde in 1× PBS at room temperature for 20 min. For DAPI staining, ovaries were washed with 1× PBS containing 0.3% Triton X-100 and mounted using ProLong Gold Antifade Mountant with DNA Stain DAPI (P36935, Invitrogen). For immunofluorescence staining, ovaries were washed with 1× PBS containing 0.3% Triton X-100 and permeabilized with 1% Triton X-100 in PBS at room temperature for 1 h. After washing with 1× PBS containing 0.3% Triton X-100, ovaries were blocked by 1% BSA in 1× PBS for 1 h and then incubated with cleaved *Drosophila* Dcp-1 (1:200 dilution; #9578, Cell Signaling) in 1× PBS at 4 °C overnight. Ovaries were washed with 1× PBS containing 0.3% Triton X-100, followed by incubation with fluorescent secondary antibody in 1× PBS at room temperature for 1 h. After extensive washing with 1× PBS containing 0.3% Triton X-100, ovaries were washed with 1× PBS and then mounted using ProLong Gold Antifade Mountant with DNA Stain DAPI (P36935, Invitrogen). Samples were observed using an inverted fluorescence microscope (Zeiss Axio Observer A1) equipped with a camera (Zeiss AxioCam MRm).

### TUNEL assay of ***Drosophila*** ovaries

*Drosophila* ovaries were dissected in cold 1× PBS, fixed with 4% formaldehyde in 1× PBS for 20 min, and then washed with 1× PBS containing 0.3% Triton X-100. Ovaries were permeabilized with 1% Triton X-100 in PBS for 1 h at room temperature and washed with 1× PBS containing 0.3% Triton X-100. TUNEL assay was performed using One-step TUNEL In Situ Apoptosis Kit (Elabscience) according to the supplier’s recommendations. Briefly, ovaries were incubated in 100 μl of TdT Equilibration Buffer at 37 °C for 30 min. After adding 50 μl Labeling Working Solution, ovaries were incubated at 37 °C for 60 min with shading light in a humidified chamber. Ovaries were washed with 1× PBS containing 0.3% Triton X-100 three times, followed by 1× PBS three times, and then mounted using ProLong Gold Antifade Mountant with DNA Stain DAPI (P36935, Invitrogen). Samples were observed using an inverted fluorescence microscope (Zeiss Axio Observer A1) equipped with a camera (Zeiss AxioCam MRm).

### Generation of oocyte-specific *Ddx3x* cKO mice

All C57BL/6 (C57BL/6JNarl) mice were purchased from the National Laboratory Animal Center in Taiwan. To generate *Ddx3x*^*loxP/loxP*^ mice, two loxP sites were placed in both intron 1 and intron 10 of the *Ddx3x* gene using a CRISPR/Cas9 nuclease-based technology (Gene Knockout Mouse Core Lab, National Taiwan University). *Zp3-Cre* (C57BL/6-Tg(Zp3-cre)93Knw/J, Stock No. 003651) transgenic mice were purchased from the Jackson Laboratory and used to excise the floxed Ddx3x sequence in oocytes specifically. *Ddx3x*^*loxP/loxP*^ females were mated with the *Zp3-Cre* transgenic males to generate *Ddx3x* cKO female mice with the genotype *Ddx3x*^*loxP/loxP*^*; Zp3-Cre*. Mice were housed in specific pathogen-free (SPF) conditions with free access to the standard rodent diet and water. The experimental procedures using mice were approved by the Institutional Animal Care and Use Committee at Chang Gung University (IACUC No. CGU110-208).

### Mouse genotyping

Mouse ear samples were lysed in lysis buffer (50 mM KCl, 1.5 mM MgCl_2_, 50 mM Tris–HCl [pH 8.5], 0.45% IGEPAL CA-630, 0.45% Tween-20, 1% proteinase K) at 56 °C for 2 h, and then heated at 96 °C for 10 min to remove proteinase K activity. Genomic DNA was extracted from mouse ear lysates and genotyped by PCR. *Ddx3x*^*loxP/loxP*^ mice were genotyped by PCR analysis using primers: 5VF1 (5′GAGCAGTGTGTAGCA TTTTTGT3′), 5VR1 (5′AGAGGTTTGAAGTCAGGGCAG3′), 3VF1 (5′AGAGAGGGAAGATCGGGTT3′), and 3VR1 (5′TAAACATCATAGTGTGGCGGA3′). *Zp3-Cre* transgenic mice were genotyped by PCR analysis using primers: Cre-F (5′GCATAACCAGTGAAACAGCATTGCTG3′) and Cre-R (5′GGACATGTT CAGGGATCGCCAGGCG3′). PCR reactions were carried out for 32 cycles (94 °C, 30 s; 55 °C, 30 s; 72 °C, 1 min).

### Mouse fertility test

*Ddx3x*^*loxP/loxP*^ and *Ddx3x*^*loxP/loxP*^*; Zp3-Cre* female mice were mated with C57BL/6 male mice (1:1), respectively. Male mice were removed 10 days after mating. The number of pups was counted. Male and female mice were randomly assigned, and the fertility test was repeated twice.

### Immunohistochemistry staining

Mouse ovaries were fixed in 10% formalin overnight at room temperature, embedded in paraffin, and then cut into 5 μm serial sections. Ovarian sections were stained with hematoxylin and eosin (H&E). For immunohistochemistry staining, ovarian sections were de-paraffinized, rehydrated, and washed in 1× PBS three times. Antigen retrieval was performed by boiling the sections for 20 min in citrate buffer (BOND Epitope Retrieval Solution 1; Leica). For quenching endogenous peroxidase activity, ovarian sections were treated with 3% H_2_O_2_ in 1× PBS at room temperature for 10 min and rinsed briefly in 1× PBS. Ovarian sections were blocked by Mouse on Mouse (M.O.M.) Blocking Reagent (Vector Laboratories) at room temperature for 1 h. Ovarian sections were rinsed in 1× PBS and incubated with mouse anti-DDX3 antibody (0.4 μg/ml; sc-365768, Santa Cruz Biotechnology) or cleaved caspase 3 antibody (1:200 dilution; #9661, Cell Signaling) at room temperature for 1 h. After washing with 1× PBS, ovarian sections were incubated with a secondary antibody at room temperature for 1 h. Amplification of antibody responses to antigen was achieved by polymeric amplification system at room temperature for 1 h. The color reaction was precipitated using diaminobenzidine (DAB) at room temperature for 10 min. Ovarian sections were stained with H&E. After washing with 1× PBS, ovarian sections were dried and mounted using Dako Mounting Medium (Agilent Technologies). Samples were observed using the Olympus Microscope Digital Camera Model DP71.

### TUNEL assay of mouse ovarian sections

Paraffin-embedded ovarian sections were de-paraffinized by xylene for 10 min twice, then hydrated with a sequential of hydrated ethanol of different percentages (100%, 95%, 90%, 80%, 75%) for 3 min each time. Ovarian sections were washed with 1× PBS for 5 min twice, then treated with 100 μg/ml proteinase K in 1× PBS at 37 °C for 20 min. Ovarian sections were washed with 1× PBS for 5 min three times. TUNEL assay was performed using the One-step TUNEL In Situ Apoptosis Kit (Elabscience) according to the supplier’s recommendations. Briefly, ovarian sections were incubated in 100 μl of TdT Equilibration Buffer at 37 °C for 30 min. After adding 50 μl Labeling Working Solution, ovarian sections were incubated at 37 °C for 60 min with shading light in a humidified chamber. Ovarian sections were washed with 1× PBS three times and then mounted using ProLong Gold Antifade Mountant with DNA Stain DAPI (P36935; Invitrogen). Samples were observed using an inverted fluorescence microscope (Zeiss Axio Observer A1) equipped with a camera (Zeiss AxioCam MRm).

### Cell culture and transfection

HeLa and 293T cells were purchased from the American Type Culture Collection (ATCC, Manassas, VA, USA). The cell lines used in this study were validated using short tandem repeat (STR) profiling and were free from mycoplasma contamination. Cells were grown in Dulbecco’s modified Eagles’ medium (DMEM) supplemented with 10% fetal bovine serum, 100 U/ml penicillin, 100 μg/ml streptomycin, and 2 mM L-glutamine at 37 °C in 5% CO_2_ incubator. Cell transfection was performed using the Lipofectamine® 2000 (Thermo Fisher Scientific) according to the manufacturer’s instructions.

### Lentivirus-mediated RNAi knockdown

The RNAi Core Facility (Academia Sinica, Taiwan) provided all of the plasmids required for lentivirus production. The two pLKO.1-shRNA vectors used to knockdown DDX3 were as follows: TRCN0000000002 (shDDX3-1) and TRCN0000000004 (shDDX3-2). The transfection reagent Lipofectamine® 2000 (Thermo Fisher Scientific) was used for lentiviral production in 293T cells with a packaging plasmid (psPAX2), an envelope plasmid (pMD2.G), and a shRNA-expressing plasmid (pLKO.1). To knock down endogenous DDX3, cells were transduced with shRNA-expressing lentivirus at a multiplicity of infection (MOI) of 5 virus particles/cell in growth medium containing 8 µg/ml polybrene at 37 °C, 5% CO_2_. After 24 h incubation, puromycin (2 μg/ml) was added to the medium for selecting infected cells. Cells were harvested 3 days after transduction for analysis.

### Quantitative real-time RT-PCR

Extracted total RNA was reverse transcribed into cDNA using the High-Capacity cDNA Reverse Transcription Kits (Thermo Fisher Scientific) according to the manufacturer’s instructions. According to the supplier’s recommendations, the resulting cDNA was subjected to quantitative real-time PCR analysis using StepOnePlus™ Real-Time PCR Systems (Thermo Fisher Scientific). Quantitative real-time PCR was performed using the Fast SYBR Green Master Mix (Thermo Fisher Scientific) and the forward and reverse primers (Table 3). The levels of mRNAs were detected by the measurement of threshold cycle (Ct) values during the exponential phase of amplification. Relative quantitation values were calculated using the 2^−ΔΔCt^ method.

**Table 3:** List of primers used for quantitative real-time PCR.

Transcript	Primer sequence
*APC1*	FP: 5′GCTCTTTATGAACTCGGAATTCCT3′
	RP: 5′AGGCGTGCACACACATATCAC3′
*CCNE1*	FP: 5′TGCTTCGGCCTTGTATCATTT3′
	RP: 5′TGGAACCATCCACTTGACACA3′
*P38γ*	FP: 5′AGGCAGGCAGACAGTGAGATG3′
	RP: 5′TGTCCACCGTCTGCGTGTAG3′
*B56β*	FP: 5′CATCCGCATGATCTCAGTGAATAT3′
	RP: 5′CCACGAAGGCTCAAGATTGG3′
*PP2Aβ*	FP: 5′TGTGCGAGAAGGCAAAGGA3′
	RP: 5′TCATGAAATTGACCATGCACATC3′
*PKACα*	FP: 5′CAAGGAGACCGGGAACCACTA3′
	RP: 5′CAGGATGCGCTTTTCATTCA3′
*FBXW11*	FP: 5′ACGCTTCAGCAATGGACTGA3′
	RP: 5′CAGGACACGGCGTAAAGTGA3′
*RAC1*	FP: 5′ATGGGATACAGCTGGACAAGAAG3′
	RP: 5′GCCCCGGGAGGTTATATCCT3′
*P38β*	FP: 5′GACCTGAACAACATCGTCAAGTG3′
	RP: 5′CGGCCGAGTGGATGTACTTC3′
*GNAS*	FP: 5′CCGGGCCAAGTACTTCATTC3′
	RP: 5′GTCCACAGCGCAGGTGAAAT3′
*DDX3*	FP: 5′CGGAGTGATTACGATGGC3′
	RP: 5′GAGTGGTTTTGACCAATCATC3′
*GAPDH*	FP: 5′CCATCTTCCAGGAGCGAGATC3′
	RP: 5′GCCTTCTCCATGGTGGTGAA3′

*FP* forward primer, *RP* reverse primer.

### Sucrose gradient sedimentation and polysome profile analysis

293T cells were collected in cold PBS containing 100 µg/ml cycloheximide. All subsequent steps were performed at 4 °C. Cell pellets were resuspended in RSB-150 (10 mM Tris-HCl [pH 7.4], 3 mM MgCl_2_, and 150 mM NaCl) containing 100 µg/ml cycloheximide, 40 µg/ml digitonin (Calbiochem), 20 U/ml RNasin (Promega), and 1× protease inhibitor cocktail (Thermo Fisher Scientific). After incubation on ice for 5 min, cells were disrupted by passage through a 26-gauge needle five times. Cytoplasmic extracts were collected by centrifugation at 3000 × *g* for 2 min and clarified by further centrifugation at 11,000 × *g* for 15 min. The samples were loaded on a linear 15-40% sucrose gradient and centrifuged at 38,000 rpm for 3 h in a Beckman SW41 rotor. After centrifugation, total RNA was extracted from each fraction using phenol/chloroform extraction in the presence of 1% SDS and 0.25 M NaCl, followed by ethanol precipitation. The translational efficiency of a transcript was calculated as previously described [21].

### Plasmid construction

For in vivo translation assay, the 5′ UTR fragments of *APC1, CCNE1, P38γ, B56β, PP2Aβ, PKACα, FBXW11, RAC1, P38β*, and *GNAS* mRNAs were obtained by RT-PCR using total RNA from 293T cells as the template and inserted into a unique *Hin*dIII site upstream of the firefly luciferase coding region in the pFL-SV40 vector [3]. All plasmid constructs were confirmed by DNA sequencing.

### In vivo translation assay

293T cells (6 × 10^4^ cells/well) were transduced with the empty lentiviral vector pLKO.1 or the pLKO.1 vector expressing shDDX3-1. At 48 h post transduction, cells were co-transfected with firefly luciferase reporter containing the 5′ UTR of DDX3 target mRNAs and the control pRL-SV40 vector encoding the *Renilla* luciferase. After 24 h post-transfection, cells were lysed in 1× Passive Lysis Buffer (Promega). The activities of firefly luciferase and *Renilla* luciferase were measured using the Dual-Luciferase Reporter Assay System (Promega).

### Statistical analysis

For each experiment, at least three independent experiments were performed. Data from independent experiments were calculated and expressed as mean ± SD. Statistical analysis was performed using one-way ANOVA or unpaired Student’s *t*-test. A *p* value < 0.05 was considered statistically significant.

## Supplementary information

Supplemental material WB

## Data Availability

Original data are available upon request.
