# Supplementary figures and images for: DDX3 is critical for female fertility via translational control in oogenesis
Source: Cell Death Discov. 2024 Nov 17;10:472. doi: 10.1038/s41420-024-02242-6 (PMC11570671; doi:10.1038/s41420-024-02242-6)

**Supplementary Figure 1. Uncropped immunoblots of the different figures.**


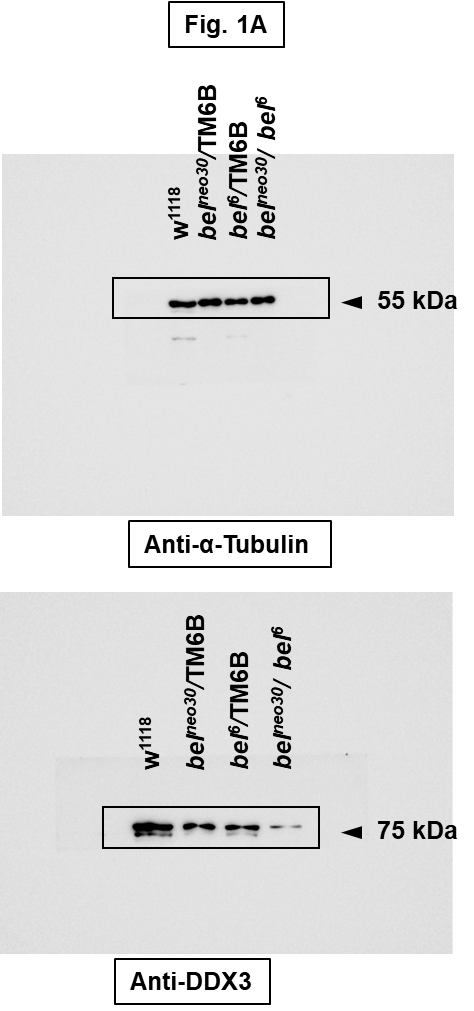


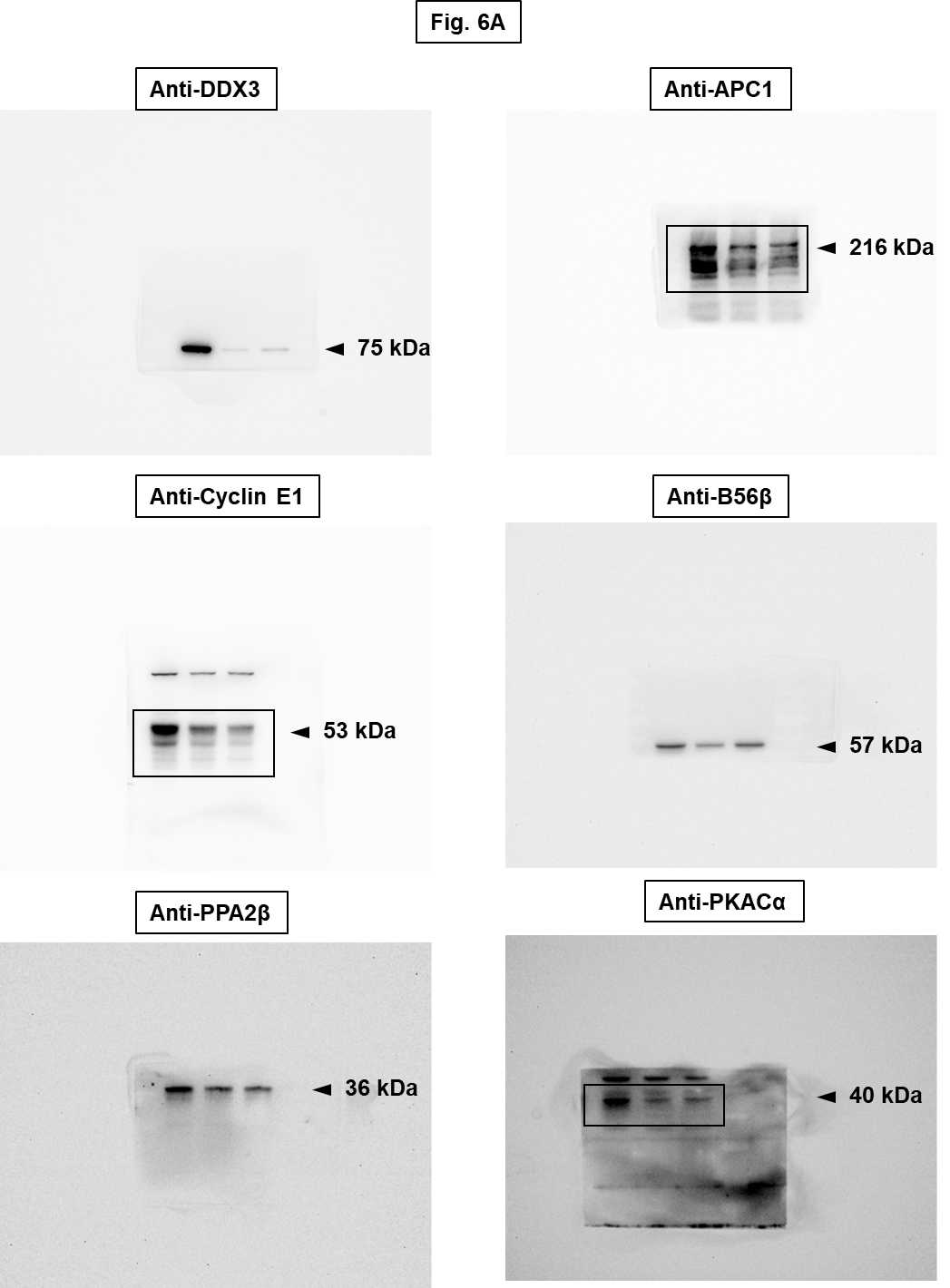


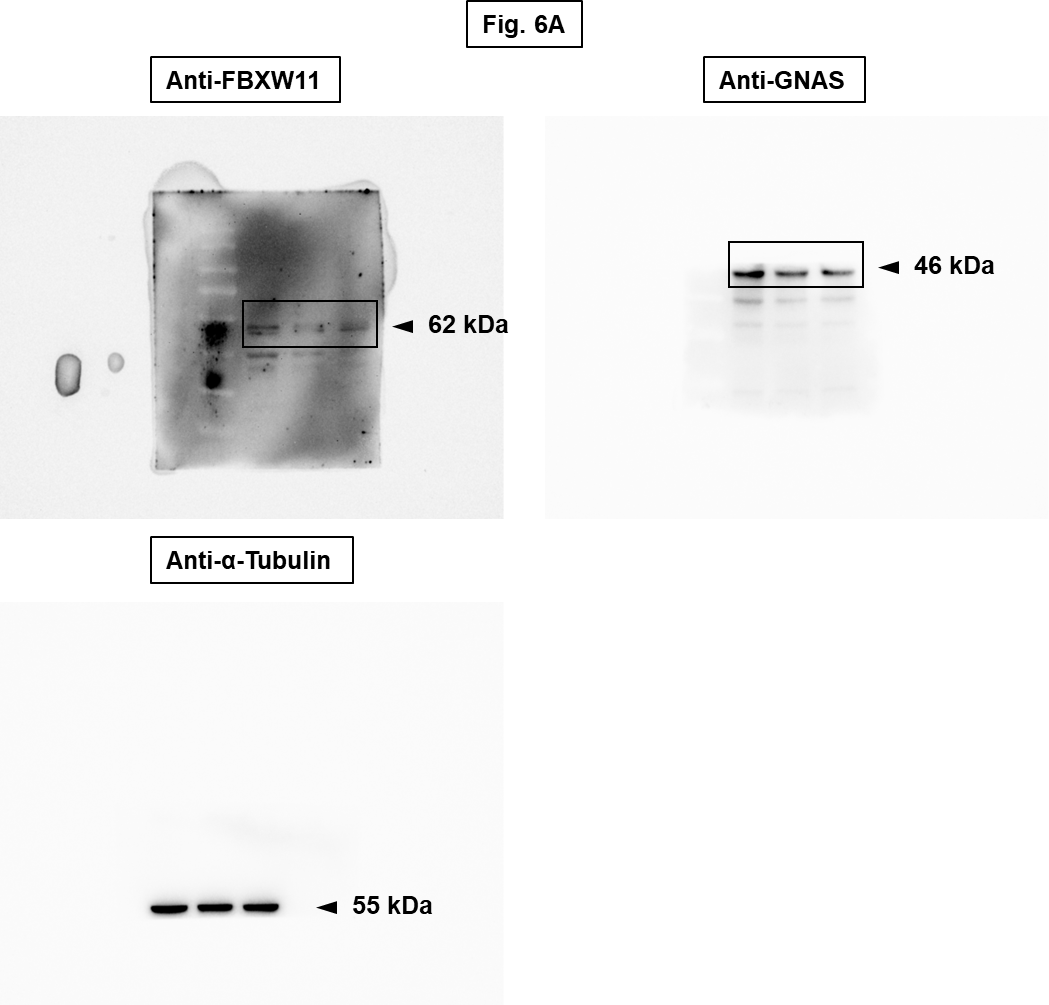

Supplement: Supplementary file 1 — Supplemental material WB [file 41420_2024_2242_MOESM1_ESM.docx]
